# Metastatic Anaplastic Lymphoma Kinase Rearrangement-Positive Adenocarcinoma of Occult Primary Mimicking Ovarian Cancer

**DOI:** 10.7759/cureus.9437

**Published:** 2020-07-28

**Authors:** Michael Chahin, Nithya Krishnan, Trevanne Matthews-Hew, Jason Hew, Dat Pham

**Affiliations:** 1 Internal Medicine, University of Florida College of Medicine – Jacksonville, Jacksonville, USA; 2 Hematology and Oncology, University of Florida College of Medicine – Jacksonville, Jacksonville, USA; 3 Hematology and Oncology, 21st Century Oncology, Jacksonville, USA

**Keywords:** alectinib, lung, adenocarcinoma, ovary, metastasis, alk inhibitor

## Abstract

Lung cancer is a worldwide concern and is the leading cause of cancer-related death in the United States. Adenocarcinoma is the most common type of non-small cell lung cancer; however, unlike other types of lung cancer this disease is often seen in light tobacco smokers and non-smokers. The presence of driver mutations, such as the epidermal growth factor receptor (EGFR) and echinoderm microtubule-associated protein-like 4 (EML-4)-anaplastic lymphoma kinase (ALK) rearrangement, appears to be more common in these patients. The presence of the ALK mutation provides a target for ALK-inhibiting agents, such as alectinib. Routine testing for driver mutations is the standard of care in the management of advanced non-small cell lung cancer.

Lung cancer frequently metastasizes to distant sites such as the bone, brain, and the adrenal glands, but rarely to the ovaries. We present a young, female, patient who complained of shortness of breath and was found to have pulmonary emboli, extensive lymphadenopathy, and a right ovarian mass. Initial pathology from a cervical lymph node favored a gastrointestinal or an ovarian malignancy. However, immunohistochemical staining ultimately suggested an occult lung adenocarcinoma primary with ovarian metastasis. She had a left oophorectomy that demonstrated similar findings and was positive for the ALK mutation. She was treated with alectinib with good response though ultimately died from her disease.

This case demonstrates the rare finding of an ALK-mutated lung adenocarcinoma with ovarian metastasis and, to our knowledge, it is the first with an occult lung adenocarcinoma primary. Driver mutation testing should be considered in metastasis from an occult primary when a pulmonary malignancy is suspected.

## Introduction

Lung cancer is the most diagnosed cancer worldwide, and the primary risk factor is tobacco consumption. Some other risk factors include exposure to certain toxins, increased age, genetic factors, and infections such as HIV [1,2]. Historically, squamous cell carcinoma was the most frequent subtype of non-small cell lung cancer (NSCLC), but newer studies show adenocarcinoma as the most common [3]. Many patients who develop adenocarcinoma of the lung have no smoking history. These patients are frequently younger and present with advanced disease. Epidermal growth factor receptor (EGFR) and echinoderm microtubule-associated protein-like 4 (EML-4)-anaplastic lymphoma kinase (ALK) mutations are often found in individuals with adenocarcinoma and no, or light (≤10 pack-years), smoking history [2,4].

An autopsy study revealed the three most common sites of hematogenous metastasis of lung cancer to be the liver, adrenal glands, and bone. Out of the 175 individuals who had lung cancer metastasis, <1% had ovarian involvement [5]. A case series demonstrated that lung adenocarcinomas with ovarian metastasis may frequently harbor the EML4-ALK mutation, thus providing a target for therapy in patients with advanced disease [6].

## Case presentation

A 29-year African American female with no smoking history presented with right lower extremity pain and swelling, and shortness of breath. She had a four-day history of productive cough without hemoptysis and a 45 pound weight loss over the past few months. She denied oral contraceptive use or tobacco use. Her physical exam was remarkable for 1+ pitting edema and tenderness to palpation of the right calf and thigh. She had right cervical adenopathy and a muffled voice. She was tachycardic but normotensive. Eastern Cooperative Oncology Group performance status was 1.

**Figure 1:**
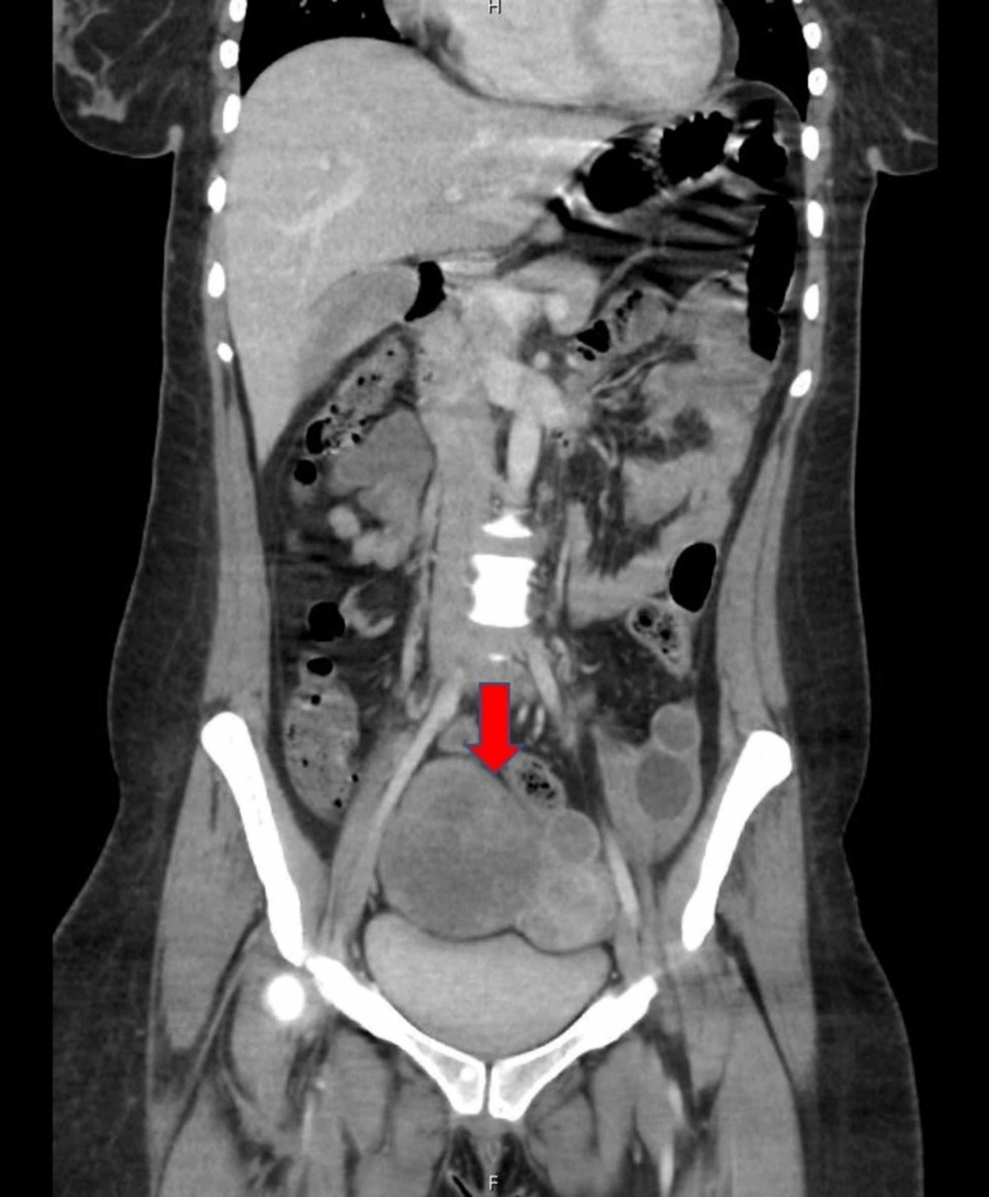
Coronal view of the abdomen and pelvis CT demonstrating a 9.5 cm solid and cystic right adnexal mass.

CT of the soft tissue neck with contrast revealed enlarged necrotic cervical, bilateral supraclavicular, and superior mediastinal lymph nodes. CT angiography showed pulmonary emboli and right peribronchial thickening without a definitive mass. A subsequent CT of the abdomen and pelvis revealed right common femoral deep venous thrombosis and a right adnexal mass (Figure 1).

**Figure 2:**
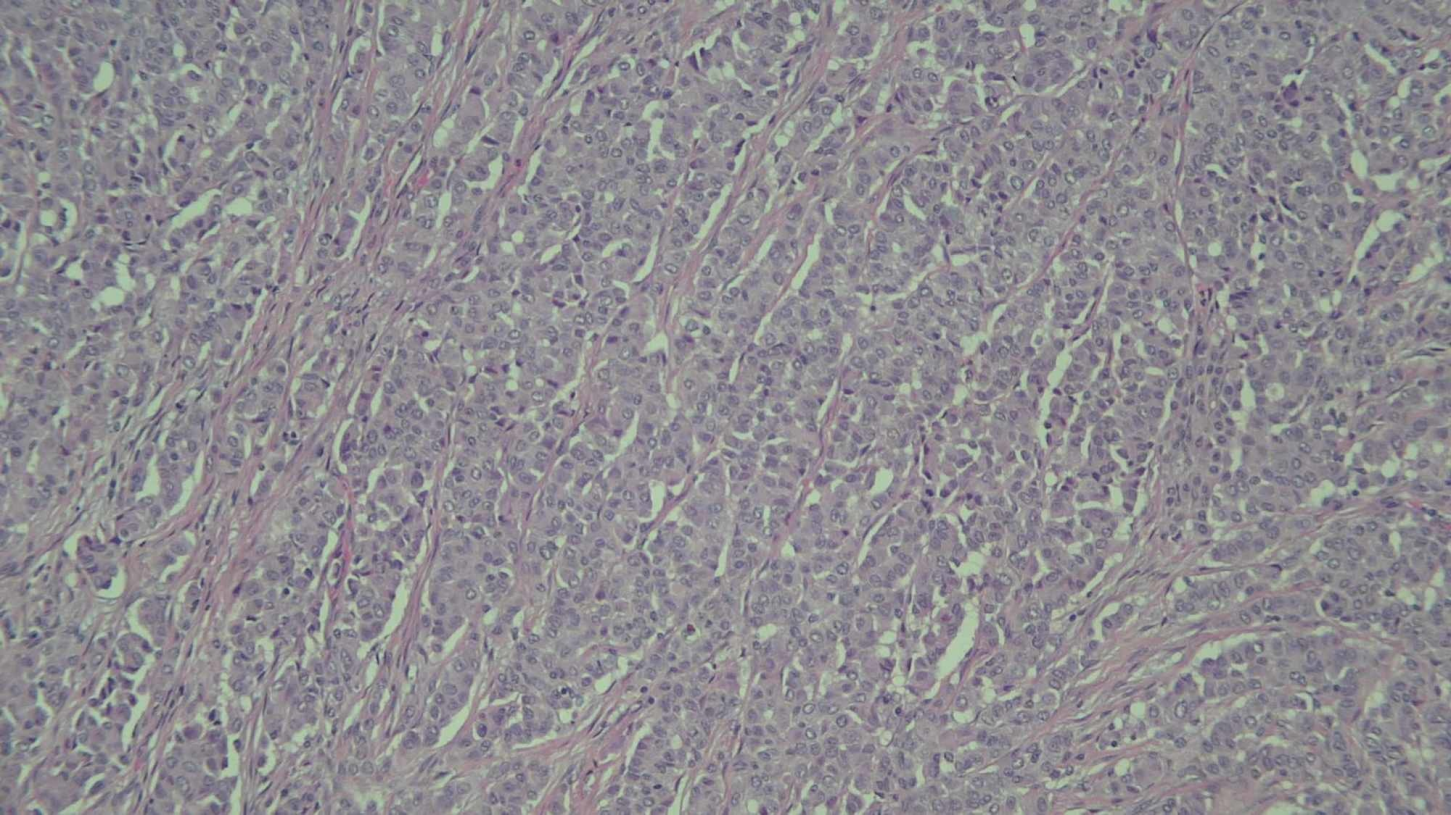
Poorly differentiated adenocarcinoma involving the ovary (H&E stain, ×100).

**Figure 3:**
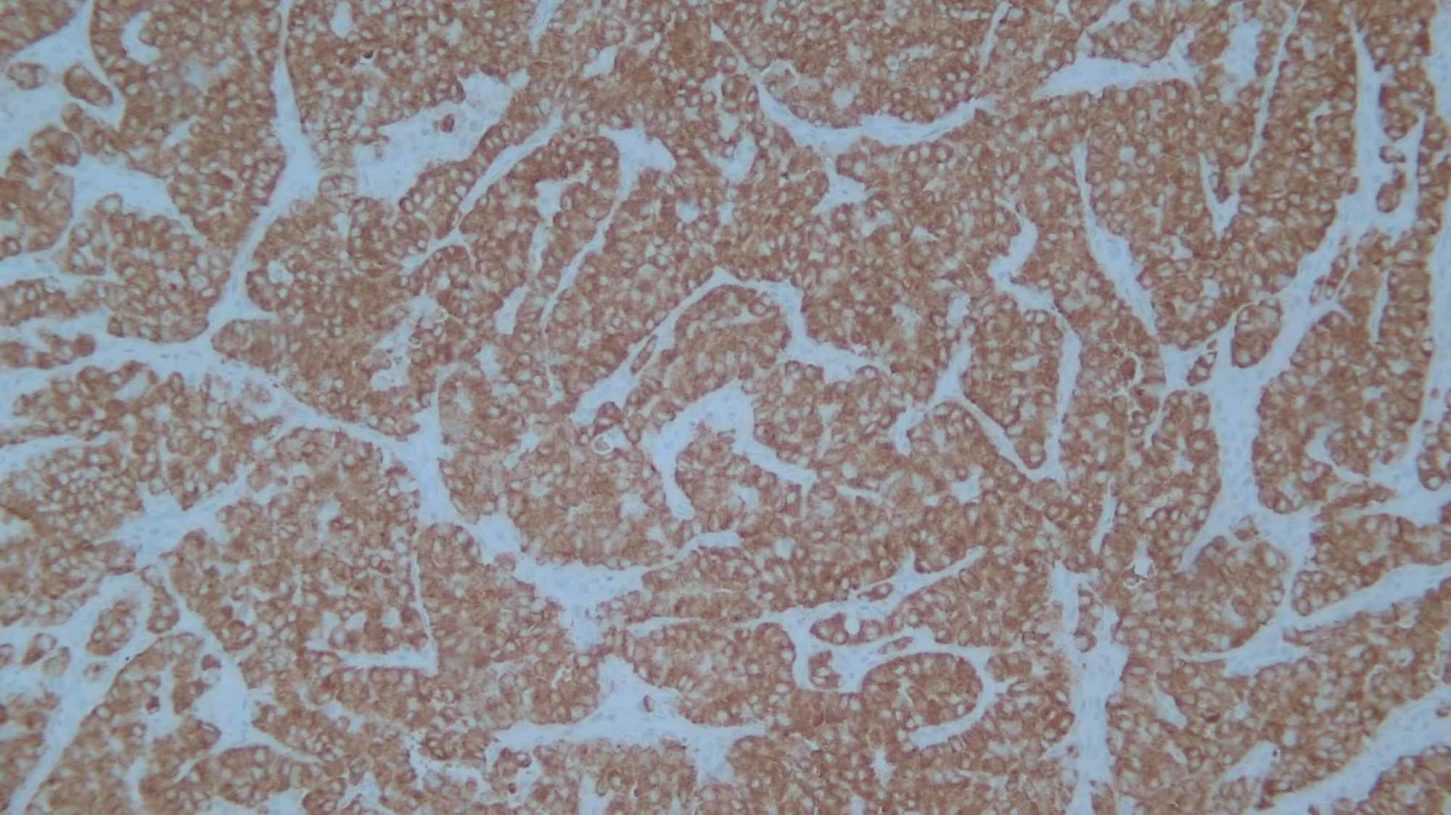
Poorly differentiated adenocarcinoma involving the ovary. The tumor cells are immunoreactive for ALK (ALK (D5F3) VENTANA FDA CDx assay, ×100). ALK, anaplastic lymphoma kinase

Biopsy of the right cervical lymph node revealed metastatic, poorly differentiated adenocarcinoma. Immunohistochemical staining showed cytokeratin (CK) AE1/AE3, CK7, thyroid transcription factor (TTF) 1, and Napsin A positivity, consistent with a lung primary. Negative immunostains included CK20, CK 5/6, thyroglobulin, calcitonin, CD56, PLAP, chromogranin, cancer antigen (CA) 19.9, CD45, and S100. She underwent left adnexal oophorectomy, from which pathology later revealed 100% ALK positive poorly differentiated adenocarcinoma of pulmonary origin, programmed cell death protein 1 (PD-1) 30% on immunohistochemical assay (Figures 2, 3).

A staging brain MRI revealed a metastatic parietal lobe lesion. After receiving stereotactic radiotherapy for brain metastasis, the patient was started on alectinib 600 mg twice daily with minimal adverse effects.

**Figure 4:**
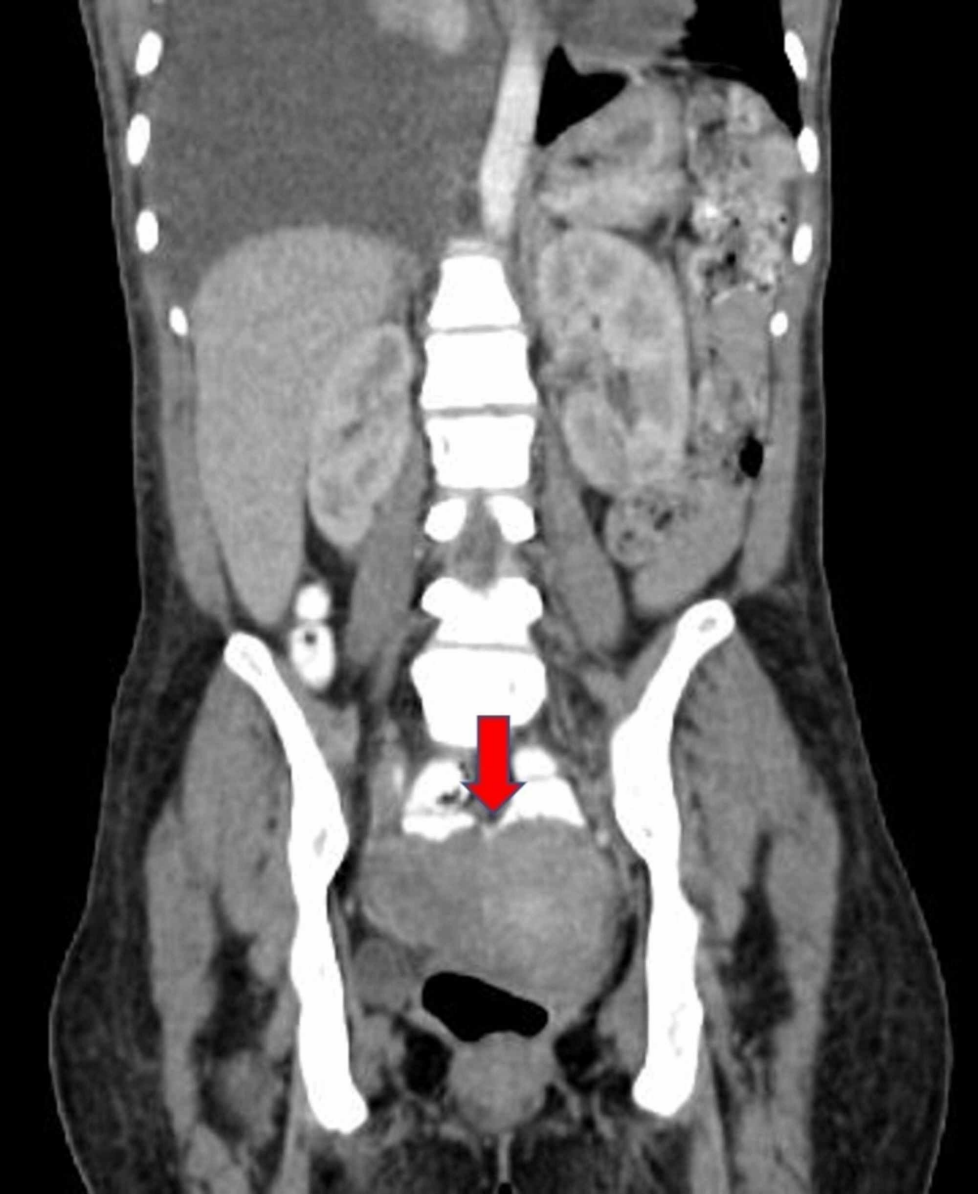
Coronal view of the abdomen and pelvis CT demonstrating a heterogeneous partially cystic right adnexal lesion, six months after starting alectinib.

Her initial CA 125 was 3,641 U/mL and carcinoembryonic antigen (CEA) was 52.1 ng/mL. After six months of therapy, her markers had decreased to 129 U/mL and 10.7 ng/mL, respectively. An initial position emission tomography (PET)/CT was not performed, but a follow-up study CT around this time revealed a slight decrease of the right ovarian mass to 7.1 cm and diffuse bony lesions (Figure 4). It was unclear if the bone lesions were new given the lack of prior imaging. 

After eight months of alectinib therapy, her CA 125 and CEA levels increased. She had increased pain from bony metastasis and required repeated therapeutic thoracentesis of a right-side malignant pleural effusion. Cytology was positive for metastatic adenocarcinoma. She was then switched to pembrolizumab, a PD-1 inhibitor, 200 mg every 21 days due to symptomatic progression of disease with multiple, systemic, lesions. Had she had been asymptomatic, had symptomatic brain recurrence only, or had an isolated lesion she would have been a candidate for continuation of alectinib.

She went on to have pathologic fracture of a thoracic vertebrae and received radiotherapy for her osseous metastases. By the time of follow-up visit three months from starting pembrolizumab, she was wheelchair-bound and requiring supplemental oxygen. The plan was to switch to brigatinib, also an ALK inhibitor, due to continued disease progression. At the time it had been approved for patients who progressed on crizotinib. Unfortunately, however, she died approximately two weeks later, prior to starting therapy.

## Discussion

On initial pathology review from the cervical lymph node biopsy, a primary ovarian malignancy and upper GI primary were strongly considered. No definitive lung mass was seen but the immunohistochemistry staining pattern was consistent with a lung primary, based on CK7, TTF1, and Napsin A positivity. 

ALK inhibitors have been shown to have improved outcomes without an increase in toxicity in comparison to chemotherapy for ALK-positive NSCLC [7]. Alectinib, a highly selective ALK inhibitor, was approved initially in 2015 for patients who had progressed on or did not tolerate crizotinib and became first line in 2017, following the alectinib versus crizotinib in untreated ALK-positive non-small cell lung cancer (ALEX) trial. Patients treated with alectinib had a response rate of 82.9%, compared to 75.5% in those treated with crizotinib [8,9]. The alectinib versus chemotherapy in crizotinib-pretreated ALK-positive non-small cell lung cancer (ALUR) trial showed higher progression-free survival in patients treated with alectinib compared to chemotherapy, 9.6 months vs 1.6 months, in patients who had progressed after platinum-based chemotherapy and crizotinib [10].

For patients who progress on an ALK inhibitor and have multiple symptomatic lesions, lorlatinib, also an ALK inhibitor, may be used as can PD-1 targeting agents such as pembrolizumab [11]. Pembrolizumab has shown promise in overall survival in NSCLC patients whose tumors express PD ligand (L)1 (>50%) [12]. At the time of this patient’s diagnosis, alectinib had only recently been approved as first-line therapy and lorlatinib was not approved in the USA at the time [13].

ALK rearrangement is found in approximately 7% of lung adenocarcinomas, compared to the EGFR mutation which is seen in approximately 15% [14,15]. The case series and literature review by Bi et al. reveals a possible association between ALK rearrangement positivity and ovarian metastasis from lung adenocarcinoma primary. This series accounts for, to our knowledge, all published cases of patients who developed ovarian metastasis from lung adenocarcinoma with ALK mutation. Out of the 16 cases evaluated in these case series, 11 of the 16 patients, or 69%, had the ALK rearrangement. Two of the 16 patients had EGFR mutation, suggesting the ALK mutation is the most common mutation in these lung cancer patients with ovarian metastasis [6]. The present case is unique from the other published reports due to this patient developing ovarian metastasis from an occult lung primary.

Patients presenting with advanced disease should have molecular testing for mutations such as ALK and PD-1 [11]. Despite having extensive disease at diagnosis, this patient had an initial positive response with alectinib. Her course was complicated by multiple hospitalizations due to shortness of breath and failure to thrive, causing delay in care. An initial PET/CT was never done, making imaging somewhat unreliable for monitoring progression. The solid portion of the ovarian mass decreased in size, but bone metastases developed. The primary objective findings in this case of good response are the tumor markers. At the time of progression of her disease her functional status had deteriorated making her not a candidate for chemotherapy.

## Conclusions

Ovarian metastasis from lung adenocarcinoma is a rare entity and seems to be associated with EML4-ALK rearrangement mutation. This case reinforces that molecular testing should be performed on all patients with systemic NSCLC. This patient presented with advanced, metastatic disease but had a good response to ALK-inhibiting therapy. 
